# Supplementary material for: Translational reading frame predicts the pathogenicity of C-terminal frameshift deletions in MeCP2
Source: eLife. 2026 Aug 10;14:RP109170. doi: 10.7554/eLife.109170 (PMC13456685; doi:10.7554/eLife.109170)
Supplement: Peer review history

# Editor report: eLife Assessment

**DOI:** 10.7554/eLife.109170.3.sa0

**Authors:** Lisa M Monteggia

**Affiliations:** Vanderbilt University United States

**Keywords:** Convincing, Important

This study offers **important** insight into the pathogenic basis of intragenic frameshift deletions in the carboxy-terminal domain of MECP2, which account for some Rett syndrome cases, yet similar variants also appear in unaffected individuals. Using base editing and mouse models, the authors present **convincing** evidence supporting the pathogenicity of select deletion variants, with potential implications for therapeutic development.

# Referee report: Reviewer #1 (Public review)

**DOI:** 10.7554/eLife.109170.3.sa1

Summary:

The authors scrutinized difference in C terminal region variant profiles between Rett syndrome patients and healthy individuals and pinpointed that subtle genetic alternation can cause benign or pathogenic output, which harbors important implication in Rett syndrome diagnosis and proposing therapeutic strategy. This work will be beneficial to clinicians and basic scientists who work on Rett syndrome and carries potential to be applied to other Mendelian rare diseases.

Strengths:

Well-designed genetic and molecular experiments, translating genetic differences into functional and clinical changes. This is a unique study resolving subtle changes in sequences give rise to dramatic phenotypic consequences.

Comments on revised version.

Improvements were made during the revision.

# Referee report: Reviewer #2 (Public review)

**DOI:** 10.7554/eLife.109170.3.sa2

Summary:

This study by Guy and Bird and colleagues is a natural follow-up to their 2018 Human Molecular Genetics paper, further clarifying the molecular basis of C-terminal deletions (CTDs) in MECP2 and how they contribute to Rett syndrome. The authors combine human genetic data with well-designed experiments in embryonic stem cells, differentiated neurons, and knock-in mice to explain why some CTD mutations are disease-causing while others are harmless. They show that pathogenic mutations create a specific amino acid motif at the C-terminus, where +2 frameshifts produce a PPX ending that greatly reduces MeCP2 protein levels (likely due to translational stalling) whereas +1 frameshifts generating SPRTX endings are well tolerated.

Strengths:

This is a comprehensive and rigorous study that convincingly pinpoints the molecular mechanism behind CTD pathogenicity, with strong agreement between the cell-based and animal data. The authors also provide a proof of principle that modifying the PPX termination codon can restore MeCP2-CTD protein levels and rescue symptoms in mice. In addition, they demonstrate that adenine base editing can correct this defect in cultured cells and increase MeCP2-CTD protein levels. Overall, this is a well-executed study that provides important mechanistic and translational insight into a clinically important class of MECP2 mutations.

Weaknesses:

The adenine base editing to change the termination codon is shown feasible in generated cell lines, but yet to be shown in vivo in animal models.

Comments on revised version.

The authors have addressed all of my questions and comments.

# Author comment: Author response

**DOI:** 10.7554/eLife.109170.3.sa3

**Authors:** Jacky Guy, Elena Hein, Beatrice Alexander-Howden, Timur von Bock und Polach, Tricia Mathieson, Ben Kleinstiver, Huda Y Zoghbi, Adrian Bird

**Affiliations:** University of Edinburgh Edinburgh United Kingdom; University of Edinburgh Edinburgh United Kingdom; University of Edinburgh Edinburgh United Kingdom; University of Edinburgh Edinburgh United Kingdom; University of Edinburgh Edinburgh United Kingdom; Massachusetts General Hospital Boston United States; Baylor College of Medicine Houston United States; University of Edinburgh Edinburgh United Kingdom

The following is the authors’ response to the original reviews.

This is a summary of the changes that have been made to the Reviewed Preprint:

(1) The data from RettBASE which was analysed in the manuscript has been added in the form of four supplementary tables. Supplementary Table 1 contains the download of all MeCP2 mutations contained in RettBASE. Supplementary Tables 2-4 contain subsets of this data which were used in Figure 2B and Figure 3 SF2. Supplementary Table 4 also has the HGVS nomenclature for both e1 and e2 isoforms and the ClinVar Variation ID for each allele. Wording has been changed to clarify that analysis in the manuscript used this data from RettBASE and not the information that was deposited in ClinVar.

(2) Similarly, Supplementary Tables 5-8 contain the gnomAD data that was used in the preparation of Figure 2, Figure 2 SF1 and Figure 3 SF1. The “high confidence” alleles in Supplementary Table 8 have been annotated with their HGVS names.

(3) The criteria for selecting “high confidence” RettBASE and gnomAD alleles have been more explicitly stated in both the Results and Materials and Methods sections.

(4) An additional “high confidence” RTT mutation (c.1152_1195) has been added to Figures 2B and 3B.

(5) Figure 3 Supplementary Figure 2 has been added to show reading frame data for all frameshifting deletions in the C-terminal deletion-prone region (CT-DPR), showing that +2 frameshifts predominate in this larger data set, not just in the “high confidence” set. This has necessitated changing the previous Fig. 3 SF2 to Fig. 3 SF3.

(6) A summary of the genetic alterations described in the manuscript, and their outcomes, has been added as Figure 7.

(7) A simple flow chart which assists in the classification of human CTDs as “likely benign” or “likely pathogenic” has been added as Figure 8. This will aid future assessment of novel mutations in this region.

(8) Additions have been made to the Materials and Methods section to comply with reporting guidelines.

(9) Minor changes have been made to the text to correct typographical errors and to clarify meaning.

**Public Reviews:**

**Reviewer #1 (Public review):**

Summary:

The authors scrutinized differences in C-terminal region variant profiles between Rett syndrome patients and healthy individuals and pinpointed that subtle genetic alternation can cause benign or pathogenic output, which harbors important implications in Rett syndrome diagnosis and proposes a therapeutic strategy. This work will be beneficial to clinicians and basic scientists who work on Rett syndrome, and carries the potential to be applied to other Mendelian rare diseases.

Strengths:

Well-designed genetic and molecular experiments translate genetic differences into functional and clinical changes. This is a unique study resolving subtle changes in sequences that give rise to dramatic phenotypic consequences.

Weaknesses:

There are many base-editing and protein-expression changes throughout the manuscript, and they cause confusion. It would be helpful to readers if authors could provide a simple summary diagram at the end of the paper.

We have added a summary diagram, as suggested (Figure 7). We have also provided a flowchart which shows how to classify human CTDs as “likely benign” or “likely pathogenic” based on their location.

**Reviewer #2 (Public review):**

Summary:

This study by Guy and Bird and colleagues is a natural follow-up to their 2018 Human Molecular Genetics paper, further clarifying the molecular basis of C-terminal deletions (CTDs) in MECP2 and how they contribute to Rett syndrome. The authors combine human genetic data with well-designed experiments in embryonic stem cells, differentiated neurons, and knock-in mice to explain why some CTD mutations are disease-causing while others are harmless. They show that pathogenic mutations create a specific amino acid motif at the C-terminus, where +2 frameshifts produce a PPX ending that greatly reduces MeCP2 protein levels (likely due to translational stalling) whereas +1 frameshifts generating SPRTX endings are well tolerated.

Strengths:

This is a comprehensive and rigorous study that convincingly pinpoints the molecular mechanism behind CTD pathogenicity, with strong agreement between the cell-based and animal data. The authors also provide a proof of principle that modifying the PPX termination codon can restore MeCP2-CTD protein levels and rescue symptoms in mice. In addition, they demonstrate that adenine base editing can correct this defect in cultured cells and increase MeCP2-CTD protein levels. Overall, this is a well-executed study that provides important mechanistic and translational insight into a clinically important class of MECP2 mutations.

Weaknesses:

The adenine base editing to change the termination codon is shown to be feasible in generated cell lines, but has yet to be shown in vivo in animal models.

This work is the obvious next step and is in progress. However, with the rise in pre- and neonatal genetic testing we felt it was important to disseminate our findings as soon as possible. The family pedigree in Figure 3C is a clear illustration of this need

**Reviewer #3 (Public review):**

Summary:

Guy et al. explored the variation in the pathogenicity of carboxy-terminal frameshift deletions in the X-linked MECP2 gene. Loss-of-function variants in MECP2 are associated with Rett syndrome, a severe neurodevelopmental disorder. Although 100's of pathogenic MECP2 variants have been found in people with Rett syndrome, 8 recurrent point mutations are found in ~65% of disease cases, and frameshift insertions/deletions (indels) variants resulting in production of carboxy-terminal truncated (CTT) MeCP2 protein account for ~10% of cases. Many of these occur in a "deletion prone region" (DPR) between c.1110-1210, with common recurrent deletions c.1157-1197del (CTD1) and c.1164_1207del (CTD2). While two major protein functional domains have been defined in MeCP2, the methyl-binding domain (MBD) and the NCoR interacting domain (NID), the functional role of the carboxy-terminal domain (CTD, beyond the NID, predicted to have a disordered protein structure) has not been identified, and previous work by this group and others demonstrated that a Mecp2 "minigene" lacking the CTD retains MeCP2 function suggesting that the CTD is dispensable. This raises an important question: If the CTD is dispensable, what is the pathogenic basis of the various CTT frameshift variants? Prior work from this group demonstrated that genetically engineered mice expressing the CTD1 variant had decreased expression of Mecp2 RNA and MeCP2 protein and decreased survival, but those expressing the CTD2 variant had normal Mecp2 RNA and protein and survival. However, they noted that differences between the mouse and human coding sequences resulted in different terminal sequences between the two common CTD, with CTD1 ending in -PPX in both mouse and human, but CTD2 ending in -PPC in human but -SPX in mouse, and in the previous paper they demonstrated in humanized mouse ES cells (edited to have the same -PPX termination) containing the CTD2 deletion resulted in decreased Mecp2 RNA and protein levels. This previous work provides the underlying hypotheses that they sought to explore, which is that the pathological basis of disease causing CTD relates to the formation of truncated proteins that end with a specific amino acid sequence (-PPX), which leads to decreased mRNA and protein levels, whereas tolerated, non-pathogenic CTD do not lead to production of truncated proteins ending in this sequence and retain normal mRNA/protein expression.

In this manuscript, they evaluate missense variants, in-frame deletions, and frame shift deletions within the DPR from the aggregated Genome Aggregated Database (gnomAD) and find that the "apparently" normal individuals within gnomAD had numerous tolerated missense variants and in-frame deletions within this region, as well as frameshift deletions (in hemizygous males) in the defined region. All of the gnomAD deletions within this region resulted in terminal amino acid sequences -SPRTX (due to +1 frameshift), whereas nearly all deletion variants in this region from people with Rett syndrome (from the Clinvar copy of the former RettBase database) had a terminal -PPX sequence, due to a +2 frameshift. They hypothesized that terminal proline codons causing ribosomal stalling and "nonsense mediated decay like" degradation of mRNA (with subsequent decreased protein expression) was the basis of the specific pathogenicity of the +2 frameshift variants, and that utilizing adenine base editors (ABE) to convert the termination codon to a tryptophan could correct this issue. They demonstrate this by engineering the change into mouse embryonic stem cell lines and mouse lines containing the CTD1 deletion and show that this change normalized Mecp2 mRNA and protein levels and mouse phenotypes. Finally, they performed an initial proof-of-concept in an inducible HEK cell line and showed the ability of targeted ABE to edit the correct adenine and cause production of the expected larger truncated Mecp2 protein from CTD1 constructs.

The findings of this manuscript provide a level of support for their hypothesis about the pathogenicity versus non-pathogenicity of some MECP2 CTT intragenic deletions and provide preliminary evidence for a novel therapeutic approach for Rett syndrome; however, limitations in their analysis do not fully support the broader conclusions presented.

Strengths:

(1) Utilization of publicly available databases containing aggregated genetic sequencing data from adult cohorts (gnomAD) and people with Rett syndrome (Clinvar copy of RettBase) to compare differences in the composition of the resulting terminal amino acid sequences resulting from deletions presumed to be pathogenic (n+2) versus presumed to be tolerated (n+1).

(2) Evaluation of a unique human pedigree containing an n+1 deletion in this region that was reported as pathogenic, with demonstration of inheritance of this from the unaffected father and presence within other unaffected family members.

(3) Development of a novel engineered mouse model of a previously assumed n+1 pathogenic variant to demonstrate lack of detrimental effect, supporting that this is likely a benign variant and not causative of Rett syndrome.

(4) Creation and evaluation of novel cell lines and mouse models to test the hypothesis that the pathogenicity of the n+2 deletion variants could be altered by a single base change in the frameshifted stop codon.

(5) Initial proof-of-concept experiments demonstrating the potential of ABE to correct the pathogenicity of these n+2 deletion variants.

Weaknesses:

(1) While the use of the large aggregated gnomAD genetic data benefits from the overall size of the data, the presence of genetic variants within this collection does not inherently mean that they are "neutral" or benign. While gnomAD does not include children, it does include aggregated data from a variety of projects targeting neuropsychiatric (and other conditions), so there is information in gnomAD from people with various medical/neuropsychiatric conditions. The authors do make some acknowledgement of this and argue that the presence of intragenic deletion variants in their region of interest in hemizygous males indicates that it is highly likely that these are tolerated, non-pathogenic variants. Broadly, it is likely true that gnomAD MECP2 variants found in hemizygous males are unlikely to cause Rett syndrome in heterozygous females, it does not necessarily mean that these variants have no potential to cause other, milder, neuropsychiatric disorders. As a clear example, within gnomAD, there is a hemizygous male with the rs28934908 C>T variant that results in p.A140V (p.A152V in e1 transcript numbering convention). This pathogenic variant has been found in a number of pedigrees with an X-linked intellectual disability pattern, in which males have a clear neurodevelopmental disorder and heterozygous females have mild intellectual disability (see PMIDs 12325019, 24328834 as representative examples of a large number of publications describing this). Thus, while their claim that hemizygous deletion variants in gnomAD are unlikely to cause Rett syndrome, that cannot make the definitive statement that they are not pathogenic and completely benign, especially when only found in a very small number of individuals in gnomAD.

We have included the possibility that mutations found in gnomAD may give rise to less severe neurological conditions in the discussion.

(2) The authors focus exclusively on deletions within the "DPR", they define as between c.1110-1210 and say that these deletions account for 10% of Rett syndrome cases. However, the published studies that are the basis for this 10% estimate include all genetic variants (frameshift deletions, insertions, complex insertion/deletions, nonsense variants) resulting in truncations beyond the NID. For example, Bebbington 2010 (PMID: 19914908), which includes frameshift indels as early as c.905 and beyond c.1210. Further specific examples from RettBase are described below, but the important point is that their evaluation of only frameshift variants within c.1110-1210 is not truly representative of the totality of genetic variants that collectively are considered CTT and account for 10% of Rett cases.

The vast majority of C-terminal truncating mutations do occur within the “CT-DPR”, likely due to its C-rich nucleotide sequence and the presence of microhomologies within the region. Looking at frameshifting deletions in RettBASE that start after the NID, a large proportion of these end within the CT-DPR and result in a -PPX ending. We decided to restrict our analysis to the c.1110-1210 region to avoid including the rarer examples that may have a different reason for their pathogenicity. We do not assert that all C-terminal truncations are pathogenic due to this mechanism, but current evidence suggests that most are.

(3) The authors say that they evaluated the putative pathogenic variants contained within RettBase (which is no longer available, but the data were transferred to Clinvar) for all cases with Classic Rett syndrome and de novo deletion variants within their defined DPR domain. Looking at the data from the Clinvar copy of RettBase, there are a number (n=143) of c-terminal truncating variants (either frameshift or nonsense) present beyond the NID, but the authors only discuss 14 deletion frameshift variants in this manuscript. A number of these variants have molecular features that do not fall into the pathogenic classification proposed by the authors and are not addressed in the manuscript and do not support the generalization of the conclusions presented in this manuscript, especially the conclusion that the determination of pathogenicity of all c-terminal truncating variants can be determined according to their proposed n+2 rule, or that all of the 10% of people with Rett syndrome and c-terminal truncating variants could be treated by using a base editor to correct the -PPX termination codon.

It is important to state here that we did not use the data in ClinVar for our analysis, but the original information that was held in RettBASE. We have clarified this in the manuscript and have now included supplementary tables containing the data we downloaded from both RettBASE and gnomAD. Table 1 contains all the RettBASE entries with *MECP2* mutations, while Tables 2-4 contain subsets of this data pertaining to CTDs. We have extended our “high confidence” set of RTT alleles to contain one more that was previously overlooked (c.1152_1195) to bring the total number of alleles to 15. Taken together these alleles account for 158 individual entries in RettBASE. We have now included an analysis of all frameshifting mutations in the CT-DPR (Supplementary Table 3, Fig. 3 SF2) which covers 69 different mutations and 260 individual entries. Of these, +2 frameshifts make up the large majority, in contrast to the gnomAD data shown in Supplementary Table 8 and Fig. 3 SF1.

(4) The HEK-based system utilized is convenient for doing the initial experiments testing ABE; however, it represents an artificial system expressing cDNA without splicing. Canonical NMD is dependent on splicing, and while non-canonical "NMD-like" processes are less well understood, a concern is whether the artificial system used can adequately predict efficacy in a native setting that includes introns and splicing.

We disagree with this opinion. We show that the loss of protein and mRNA seen with knock in mouse and human alleles is recapitulated when using a cDNA-based transgene in the HEK system, demonstrating that the mechanism of loss does not involve factors bound at splice junctions etc. We also demonstrate the effect of the A to G change at the stop codon is the same whether we do this by base editing our cDNA transgene in T-REx cells or by making the CTD1 X>W knock-in mouse. Both result in increased levels of a slightly extended but still truncated protein.

**Recommendations for the authors:**

**Reviewer #1 (Recommendations for the authors):**

The phrase in the title, "an alternative therapeutic approach" is only insinuated in the manuscript, making it rather inappropriate to be in the title.

In this study we use adenine base editing to modify RTT-causing CTD mutations in T-REx cells which is clearly a precursor to developing a therapy, utilising the new findings in this study. We therefore feel that the use of “an alternative therapeutic approach” can be justified.

**Reviewer #2 (Recommendations for the authors):**

I have a few minor comments for the authors to consider:

(1) Please double-check Figure 2 Supplementary Figure 1, as the allele count for E394K does not appear to be in the thousands; rather, E397K seems to be the variant shown in the graph.

Yes, this was an error and has been corrected to E397K in the text. Thank you for spotting it.

(2) On page 12, the phrase “common DNA sequence features shared by all CTDs that give rise to RTT” might be better described as “amino acid sequence features.”

This has been altered in the text as suggested.

(3) On page 3, the sentence "analysis of patient mutations and experimental data from mouse models support a role in transcriptional repression" cites Gabel et al. 2015 and Kinde et al. 2016, which focus on null alleles but not patient mutations. It would be appropriate to also cite Johnson et al. 2017, which analyzed MeCP2 T158M and R106W patient mutations.

This has now been cited.

(4) In the same sentence, Bajikar et al. 2025 are described as studying the "acute loss of MeCP2," but gene expression was analyzed after a week or longer period of time, not minutes to hours as in degron-mediated degradation systems.

The word “acute” has been removed from the text.

(5) On page 7, the statement that "E394K is common... who were later found to have additional pathogenic MECP2 mutations" should include a supporting reference.

We have now included the references Moncla et al (2002) and Wan et al (1999) to address this.

(6) Similarly, on page 10, the sentence "these findings question the validity of two cases where individuals presented with classical Rett..." is missing a reference to the case report mentioned.

The references Bienvenu et al (2000) and Philippe et al (2006) have been added.

(7) While the manuscript is well written and full of detail, if space is an issue, the authors might consider tightening sections that reiterate findings from their 2018 HMG paper.

A section discussing the CTD2 allele from the 2018 HMG paper has been removed from the results section.

**Reviewer #3 (Recommendations for the authors):**

(1) Overall, the manuscript is rather dense and potentially challenging to follow easily, especially for a non-expert reader.

Minor edits have been made to the text which will hopefully make it easier to follow. We have also added two new figures (Figures 7 and 8) to summarize the different alleles and edits which appear in the paper, and to show how to determine whether a CTD in the region is likely to be benign or pathogenic.

(2) The introduction of data presented in Figure 1 within the manuscript introduction seems inappropriate and should be moved to the results section.

We would say that this is unconventional rather than inappropriate, and is referred to in the introduction, so we would prefer to leave it as it is.

(3) Providing specific, common nomenclature for genetic repository variants (rs numbers, gnomAD IDs, etc) somewhere would be beneficial. This is an issue because of the complexity of numbering (either coding or protein) for MECP2 due to the different transcript-based numbering systems.

This nomenclature is now included in the supplementary tables of data from RettBASE and gnomAD.

(4) As described in the public comments, there are a number of MECP2 genetic variants listed in the Clinvar copy of RettBase, resulting in c-terminal truncations that are not mentioned or discussed within the manuscript. Without the level of detail present in the original form of RettBase (number of events, de novo, etc) in the currently available Clinvar iteration, it is unclear why a number of variants, even within the limited DPR region, were not mentioned. A supplementary file including the more complete information from the RettBase version, with a complete listing of all c-terminal truncating variants, and an explicit rationale for the exclusion of variants would be helpful.

We have now included supplementary tables with our download of all *MECP2* mutations which were held in RettBASE. We have further added tables with the subset of mutations that we have analysed and have more explicitly stated our criteria for defining the “high confidence” sets of mutations. We did not download the data relating to “evidence of pathogenicity” (ie de novo?, absent from parents etc) from RettBASE, but annotated our list of CTDs with this information while RettBASE was still available. This was used in Supplementary Table 3.

(5) A discussion of the limitations, notably that the fact that the focus exclusively on deletion variants within a restricted region (c.1110-1210) does not truly represent all genetic variants that cumulatively account for 10% of Rett cases, is needed. Furthermore, as pointed out, not all frameshift variants, even those that are n+2, result in the -PPX termination that is presented as the pathogenic basis of c-terminal truncations and amenable to correction by ABE. This should be noted in the discussion, as well as consideration of the late nonsense variants that cause c-terminal truncations (some of which would be very similar to the deletion variants discussed but without the proposed primary pathogenic driver, -PPX).

We do not claim to explain the pathogenic mechanism of all C-terminal frameshift mutations found in cases of Rett syndrome. There will certainly be some that do not fit our explanation. However, we believe we have shown evidence that a large proportion of CTDs in RTT will be amenable to the therapy we propose.

(6) Regarding point 3 in the public review, specifically:

(a) n=7 nonsense variants (S360X, K363X, E397X, R453X, E455X) that do not carry the destabilizing -PPX sequence.

(b) n=136 frameshift indel variants beyond the NID.

(i) n=11 that have indels that extend past the native stop codon, n=4 of which start within the DPR domain (c.1110-1210) but would have a different terminal sequence than their proposed pathogenic -PPX sequence.

(ii) n=125 frameshift indels with terminal breakpoint before the native stop codon

(c) n=89 that have start or stop points within c.1110-1210

(d) n=72 not mentioned within the manuscript.

(e) n=27 are n+1, with 26/27 having what the authors term as the "tolerated" -SPRTX ending, but 1/27 having a frameshift beyond this region (c.1133_1361)

(f) n=45 are n+2, with 32/45 ending in -PPX (supporting authors conclusion), but 10/45 will use the frameshift stop codon preceding the -PPX and have a different terminal sequence, and 3/45 result in a frameshift termination beyond the -PPX sequence.

(g) n=36 have breakpoints either before c.1110 or after c.1210

(h) n=16 start before c.1110, with 11/16 ending before c.1110. 4 of these 11 are n+2, but would use the earlier frameshift stop codon and not have -PPX terminal sequence. For 5/16, the indel extends past c.1210, with the n+2 leading to frameshift termination codons beyond the -PPX sequence.

(i) n=20 indels start beyond c.1210, with 8/20 being n+1 and 12/20 being n+2, with neither leading to the -SPRTX or --PPX termination sequences characterized in this manuscript.

As mentioned previously, we have used data taken from RettBASE, not from ClinVar. Both RettBASE and ClinVar will contain *MECP2* mutations found in cases of RTT which are not the causative mutation. Databases of this kind contain sequencing errors and mutations that have been mistakenly assigned as causative. It is therefore imperative that the publicly available information is screened to only include mutations that meet stringent criteria. This is why we chose to start by looking at high confidence sets of mutations, with our conclusions supported by analysis of all such mutations in our region of study.

As mentioned in response to point 5 above, we do not claim to explain every mutation in the region, but believe this study reveals an important disease mechanism for a large proportion of CTDs, leading to a potential therapy. It also contains significant information for predicting the likely prognosis of individuals with CTDs, who may remain healthy but are currently informed that their mutation is likely pathogenic. At present it seems that this is often based solely on the presence of a frameshifting mutation with similarities to *bona fide* RTT CTDs, without strong evidence of pathogenicity.
